# Microwave ablation induces abscopal effect *via* enhanced systemic antitumor immunity in colorectal cancer

**DOI:** 10.3389/fonc.2023.1174713

**Published:** 2023-04-27

**Authors:** Lu Yu, Hairong Xie, Linping Wang, Min Cheng, Jie Liu, Jiamei Xu, Zhigang Wei, Xin Ye, Qi Xie, Jing Liang

**Affiliations:** ^1^ Department of Oncology, The First Affiliated Hospital of Shandong First Medical University and Shandong Provincial Qianfoshan Hospital, Shandong Key Laboratory of Rheumatic Disease and Translational Medicine, Shandong Lung Cancer Institute, Jinan, China; ^2^ Department of Oncology, Feicheng People’s Hospital, Feicheng, China; ^3^ Department of Gerontology, The First Affiliated Hospital of Shandong First Medical University and Shandong Provincial Qianfoshan Hospital, Jinan, China; ^4^ School of Clinical Medicine, Weifang Medical University, Weifang, China

**Keywords:** microwave ablation, abscopal effect, colorectal cancer, immune microenvironment, T cells

## Abstract

**Background:**

Thermal ablation is the primary procedure for the local treatment of lung metastases. It is known that radiotherapy and cryoablation can stimulate an abscopal effect, while the occurrence of abscopal effect induced by microwave ablation is less; the cellular and molecular mechanisms involved in the abscopal effect after microwave ablation should be further elucidated.

**Methods:**

CT26 tumor-bearing Balb/c mice were treated with microwave ablation with several combinations of ablation power and time duration. The growth of primary or abscopal tumors and the survival of mice were both monitored; moreover, immune profiles in abscopal tumors, spleens, and lymph nodes were examined by flow cytometry.

**Results:**

Microwave ablation suppressed tumor growth in both primary and abscopal tumors. Both local and systemic T-cell responses were induced by microwave ablation. Furthermore, the mice exhibiting significant abscopal effect after microwave ablation markedly elevated Th1 cell proportion both in the abscopal tumors and spleens.

**Conclusions:**

Microwave ablation at 3 w–3 min not only suppressed tumor growth in the primary tumors but also stimulated an abscopal effect in the CT26-bearing mice *via* the improvement of systemic and intratumoral antitumor immunity.

## 1 Introduction

In the course of a person’s lifetime, approximately 4%–5% of people will develop colorectal cancer (CRC), and up to 20% of those cases have distant metastasis at the time of initial diagnosis (1, 2). The lung is one of the most common metastatic sites of CRC, affecting approximately 27% of patients with CRC (3). The 5-year survival of patients with distant disease is approximately 12% (4). While surgery is a common option for treating lung metastasis, ablation has emerged as a superior option that can retain more lung tissue and function while removing tumors (5). On the other hand, stereotactic ablative radiotherapy (SABR) or thermal ablation has been the optimized procedure for the local treatment of lung metastases (6). Microwave ablation (MWA) is one type of localized thermal ablation that stimulates coagulation necrosis through the movement of charged particles and polar molecules, and it is now the major treatment for liver and lung metastases of CRC (6).

Microwave ablation (MWA) has several advantages over radiofrequency ablation (RFA), such as better heating of larger tumors and faster and more efficient heating, which reduce sensitivity to heat sink effects. MWA is particularly suitable for tissues with higher impedance, including the lung and bone, and high water content, such as solid organs and tumors (7). The lung microwave radiofrequency (LUMIRA) randomized trial showed that MWA resulted in less intraoperative pain and significantly reduced tumor volume compared to RFA treatment (8). Retrospective analysis suggested that percutaneous MWA is a potentially safe and effective approach for treating lung malignancies, and it may enhance survival in patients who are not surgical candidates (9).

The abscopal effect is a phenomenon in radiotherapy, which is characterized by the reduction in tumor volume outside the radiation range. Mole first described it and found that the volume of untreated tumors declined following radiotherapy for primary tumors or metastatic tumors (10). There is increasing evidence that local radiotherapy can stimulate systemic antitumor effects, with tumor regression at the distal non-irradiated site, i.e., the abscopal effect of radiotherapy (11, 12). The abscopal effect of cryoablation has also been shown that distant metastases like the spine, lung, and supraclavicular lymph nodes disappeared following cryoablation of primary prostate cancer (13). Additional research has revealed that cryoablation stimulated strong and complex immune responses and activated innate and adaptive immunity (14). Conversely, there are few reports on the abscopal effect of MWA.

In clinical work, our team discovered that the abscopal effect occurred in a patient with refractory multiple pulmonary metastases of endometrial carcinoma following MWA. MWA was conducted on one side of the lung metastases, while other lung metastases disappeared, suggesting that MWA stimulated the perfect abscopal effect (15). We also observed an abscopal effect in another case presenting lung adenocarcinoma with multiple metastases in both lungs. Two nodules in the right lung were treated with MWA, and all nodules in both lungs disappeared after 2 months of Camrelizumab treatments, verifying that the abscopal effect has been stimulated by combinational therapy of MWA and PD-1 inhibitor (16). Nevertheless, not all CRC patients who were treated for lung metastases following MWA induced the abscopal effect, and the immune response stimulated by MWA in CRC is not fully elucidated. Therefore, the study aimed to investigate the immune profiles and the cellular mechanism of the abscopal effects induced by MWA in CRC.

## 2 Materials and methods

### 2.1 Cell line

CT26 cell line was procured from the Chinese Academy of Sciences (Shanghai, China). The cell culture medium comprised of Roswell Park Memorial Institute 1640 culture medium (RPMI 1640, Gibco, Cat. No. C11875500BT), 10% fetal bovine serum (FBS, Gibco, Cat. No.10270-106), and 1% streptomycin and penicillin. The cells were regulated at 37°C in a humid atmosphere containing 5% CO_2_.

### 2.2 Animal models

Seven-week-old female Balb/c mice (Vital River Laboratories, Beijing, China) were utilized and housed in the animal experiment center of the First Affiliated Hospital of Shandong First Medical University. CT26 cells (1 × 10^6^) in 100 μl of phosphate-buffered saline (PBS) were injected subcutaneously into the groin of mice to develop a primary tumor. Next, 2 × 10^5^ CT26 cells in 100 μl of PBS were injected subcutaneously to construct the abscopal tumor (17). Tumor diameters were measured every 3 days, and tumor volume was computed with the formula V= 0.5 × (A × B^2^), where A and B are the longer and shorter diameters of the tumor. Experiments were carried out under a project license (No. S1051) granted by the institutional ethics board of the First Affiliated Hospital of Shandong First Medical University and Shandong Provincial Qianfoshan Hospital, following institutional guidelines for the care and use of animals in the First Affiliated Hospital of Shandong First Medical University and Shandong Provincial Qianfoshan Hospital.

### 2.3 Study design

Animal models were constructed for various purposes. First, to determine the suitable power and time of MWA to suppress tumor growth in CT26 tumor-bearing mice, the animal model was constructed following the following procedures. CT26 cells (1 × 10^6^) in 100 μl of PBS were injected subcutaneously into the groin of mice to construct a primary tumor. When the tumor volume reached 250–500 mm^3^, the tumor-bearing mice were randomly split into seven groups (n = 4 in each group), which were treated with MWA at various power and time combinations, respectively. The tumor volume and survival status of the mice were detected, and the mice were sacrificed on day 7. Second, we developed a tumor model to investigate whether MWA induced the abscopal effect. To establish a primary tumor, we injected 1 ×106 CT26 cells in 100 μl of PBS subcutaneously into the groin of mice. Then, we constructed an abscopal tumor by injecting 2× 105 CT26 cells in 100 μl of PBS subcutaneously (17). When the *in situ* tumor volume reached 250–500 mm^3^, we randomly divided the tumor-bearing mice (n = 6 in each group) into two groups. The non-ablation group served as the control. Flow cytometry was used to analyze immune cells in tumors, spleens, and axillary lymph nodes. Third, to investigate the mechanism underlying the differential induction of the abscopal effect in mice, we constructed a CT26 tumor-bearing mouse model similar to that described in Part 2. The mice were divided into an untreated group and an MWA group (3 w–3 min) with n=9 and n=18, respectively. Tumor volume measurements of the abscopal tumors were taken every 2 days until the 10th day. Subsequently, scatter plots and linear trendlines were generated using Excel according to the tumor volumes. Nine mice exhibiting a more significant abscopal effect were defined based on the smaller slopes of their tumors (No. 2, 3, 5, 6, 7, 8, 9, 11, 17). An additional nine mice in the MWA group with larger slopes were defined as the abscopal effect group (No. 1, 4, 10, 12, 13, 14, 15, 16, 18). Based on the growth rate of the abscopal tumors, the MWA group was further divided into two groups: the more significant abscopal effect group (n=9) and the abscopal effect group (n=9). On day 10, the mice were sacrificed, and changes in immune cell populations in the tumor and spleen were observed by flow cytometry (see **Supplementary Figure S1**).

### 2.4 Microwave ablation

The primary tumor was treated with MWA using a microwave generator (Canyon Medical, Nanjing, China, KY-2000) when the tumor diameter reached 8–10 mm. Mice were anesthetized using intraperitoneal injection of avertin (1.25% avertin, EasyCheck, Nanjing AIBI Bio-Technology Co. Ltd., M2910). MWA antenna (Canyon Medical, Nanjing, China, KY-2450A-1) was inserted into the center of the primary tumor and treated with MWA with various powers and times. The microwave radiation frequency used in our experiments was 2,450 MHz.

### 2.5 Antibodies and reagents

The following reagents were utilized in the experiments: PE anti-mouse CD11c (Biolegend, Cat. No. 117308, USA), APC anti-mouse CD86 (Biolegend, Cat. No. 105008, USA), FITC anti-mouse CD4 (Biolegend, Cat. No. 100406, USA), APC anti-mouse CD8a (Biolegend, Cat. No. 100712, USA), PE anti-mouse IFN-γ (Biolegend, Cat. No. 505808, USA), and PE anti-mouse TNF-α (Biolegend, Cat. No. 506306, USA).

### 2.6 Flow cytometric analysis

The tumors, spleens, or draining lymph nodes were sheared, while tumors were further digested by a Tumor Dissociation Kit (Miltenyi, Cat No.130-096-730). Then, the cell mixture was passed through 70-μm nylon mesh cell strainers (WHB-70 μm) to obtain a single-cell suspension. Erythrocytes were extracted with cell lysis buffer (BD Biosciences, Cat. No. 20180816). To perform intracellular cytokine staining, the obtained cells were activated with eBioscience™ Cell Stimulation Cocktail Plus Protein Transport Inhibitors (Invitrogen, Cat. No. 00-4975-93) for 6 h. The single-cell suspension was stained with viability dye (Zombie violet fixed active kit, Biogene, Cat. No. 423114) to differentiate dead cells from living cells. Afterward, the percentages of various immune cells in the tumor or spleen were identified using a FACS flow cytometer (BD FACSAria™ III Cell Sorter, BD Biosciences, San Jose, CA, USA) and examined by FlowJo software.

### 2.7 Statistical analysis

GraphPad Prism software was utilized for statistical analysis, and the data were expressed as mean ± SD. One-way ANOVA was employed to analyze the significance of the differences among the three groups. The unpaired Student’s t-test was utilized for the significance analysis between the two experimental groups. The survival rate was evaluated by the Kaplan–Meier method. p < 0.05 were considered to be statistically significant.

## 3 Results

### 3.1 MWA at 3 w–3 min exhibited better antitumor efficacy but less toxicity in CT26 tumor-bearing mice

**Figure 1:**
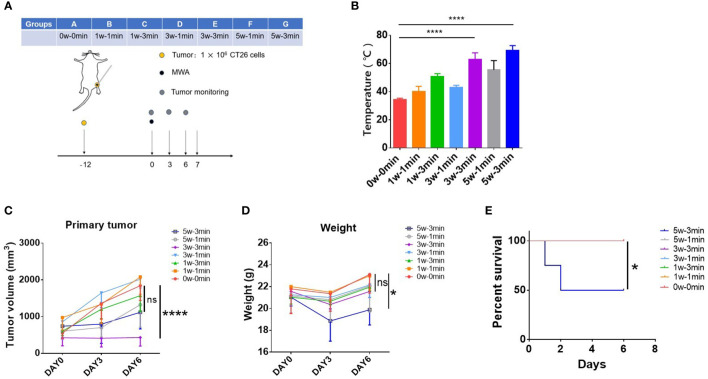
MWA at 3 w–3 min exhibited better antitumor efficacy but less toxicity in CT26 tumor-bearing mice. **(A)** Balb/c mice were inoculated with CT26 cells. Tumors were treated with MWA (n = 4 in each group) when the tumor size reached 250–500 mm^3^ in volume. **(B)** The temperature of tumors was determined following MWA. ****p < 0.0001. **(C)** The growth of the tumor was observed during the observation period. ****p < 0.0001; ns, p > 0.05. **(D)** The weight of tumor-bearing mice after MWA treatment was determined during the observation period. *p<0.05; ns, p > 0.05. **(E)** Kaplan–Meier curves illustrated the survival of mice treated with MWA with different ablation powers or times. *p < 0.05.

To determine the suitable power and time of MWA to suppress tumor growth in CT26 tumor-bearing mice, mice were treated with MWA at various conditions (0 w–0 min, 1 w–1 min, 1 w–3 min, 3 w–1 min, 3 w–3 min, 5 w–1 min, and 5 w–3 min) (**Figure 1A**). Compared with the control (34.48 ± 0.8617°C), the temperature of tumors treated with MWA at 3 w–3 min (62.88 ± 4.641°C) (p < 0.0001) or 5 w–3 min (69.23 ± 3.428°C) (p < 0.0001) was significantly higher (**Figure 1B**). Tumor growth in 3 w–3 min group was significantly suppressed following MWA in comparison with the control group (p < 0.0001, **Figure 1C**).

The weight and survival of mice were both supervised to examine the toxicity of MWA treatment in CT26 tumor-bearing mice. The weight of mice treated with MWA at 5 w–3 min decreased significantly on the third day, although it gradually gained on day 6 (19.885 ± 1.393 g) (p < 0.05, **Figure 1D**). Two mice died in MWA at 5 w–3 min group on days 1 and 2 after MWA treatments (**Figure 1E**), suggesting that MWA at 5 w–3 min was more traumatic than MWA at 3 w–3 min. According to the above results, MWA at 3 w–3 min was chosen for the subsequent experiments, as it portrayed better antitumor efficacy but less toxicity in CT26 tumor-bearing mice.

### 3.2 Abscopal effect was significantly induced by MWA at 3 w–3 min in CT26 tumor-bearing mice

**Figure 2:**
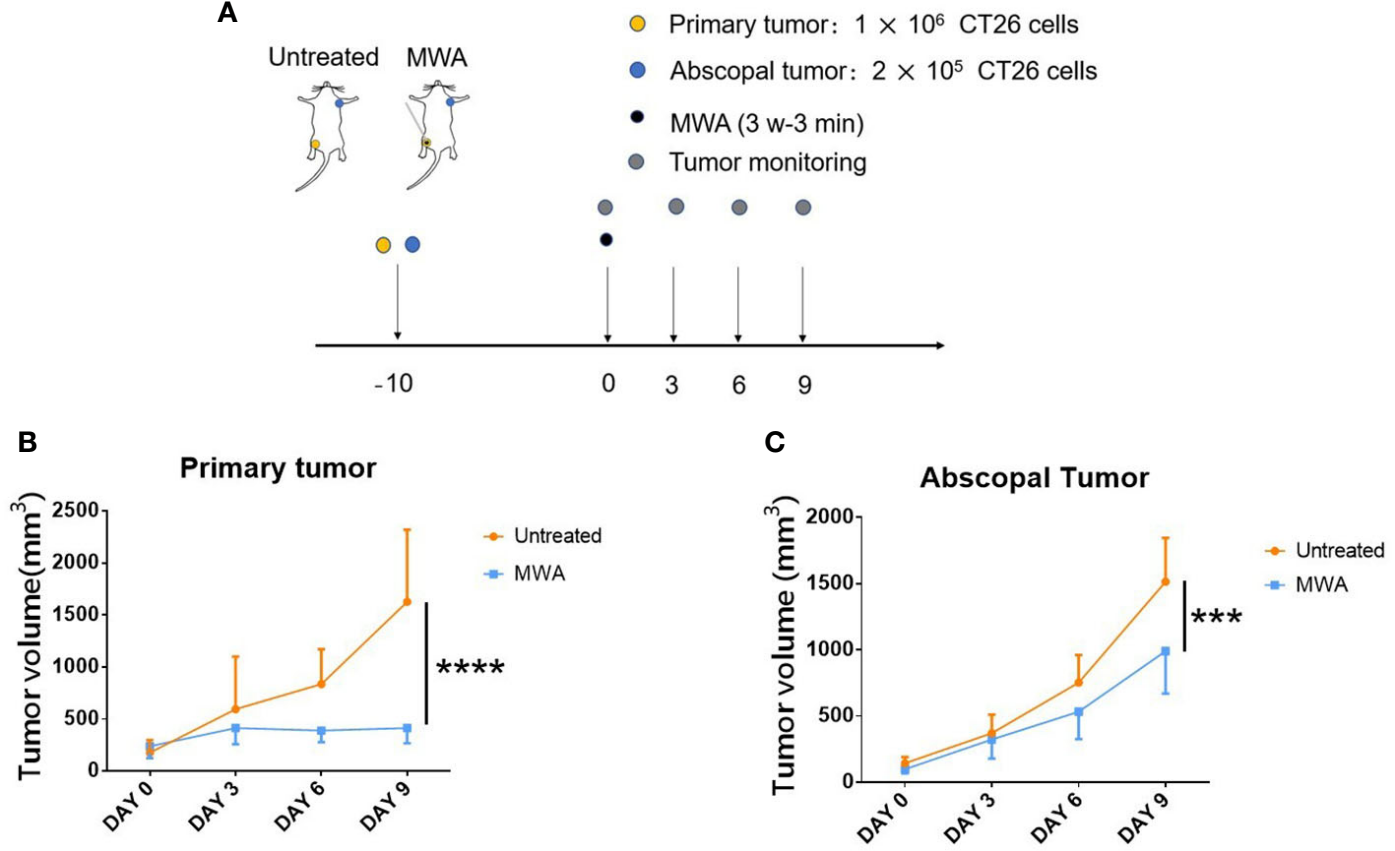
Abscopal effect in CT26 tumor-bearing mice was significantly induced by MWA at 3 w–3 min. **(A)** Balb/c mice were inoculated with CT26 cells. When the tumor size attained 250–500 mm^3^ in volume, tumor-bearing mice were treated with MWA (n = 6 in each group). **(B)** The growth of primary tumors was identified after MWA during the observation period. ****p < 0.0001. **(C)** The growth of abscopal tumors was determined following MWA during the observation period. ***p < 0.001.

The mice model was constructed following previous research to determine whether the abscopal effect was induced after MWA (18). Specifically, 1 × 10^6^ CT26 cells were injected subcutaneously to activate a primary tumor in the right groin, and 2 × 10^5^ CT26 cells were injected subcutaneously to simulate the abscopal tumor in the left axilla. MWA was carried out when the volume of the primary tumor reached 250–500 mm^3^ (**Figure 2A**). The primary tumor was completely ablated after MWA and gradually carbonized (**Figure 2B**). There was a gradual increase in the difference between the mean volume of abscopal tumors in the MWA group and the control group. On day 9 after MWA, the abscopal tumor volume in the MWA group (990.167 ± 321.343 mm^3^) was less than that in the control group (1513.833 ± 331.515 mm^3^) (p < 0.001, **Figure 2C**), suggesting that MWA at 3 w–3 min activated the abscopal effect in CT26 tumor-bearing mice.

### 3.3 MWA at 3 w–3 min activated systemic immune responses in CT26 tumor-bearing mice

**Figure 3:**
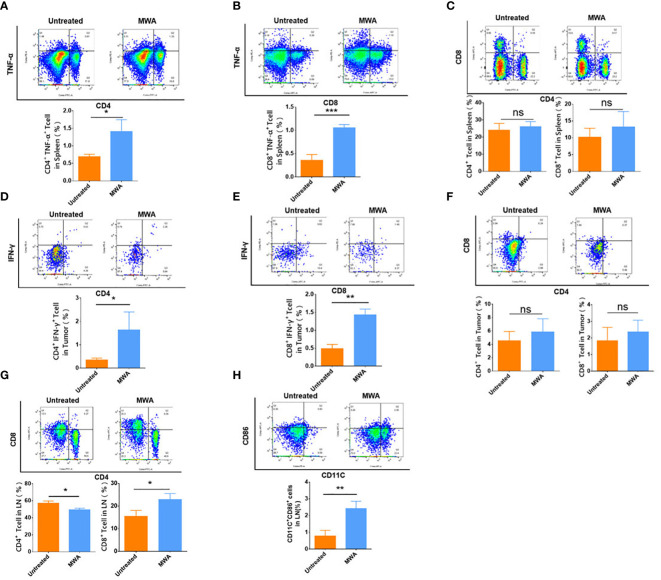
MWA at 3 w–3 min activated systemic immune response in CT26 tumor-bearing mice. The percentages of CD4^+^ TNF-α^+^ T-cells **(A)** and the percentages of CD8^+^ TNF-α^+^ T cells **(B)** in the spleen were both determined after MWA. **(C)** The percentages of CD4^+^ and CD8^+^ T cells in the spleen were determined after MWA. The percentages of CD4^+^ IFN-γ^+^ T cells in the abscopal tumor **(D)** and CD8^+^ IFN-γ^+^ T cells in the abscopal tumor **(E)** were determined after MWA. **(F)** The percentages of CD4^+^ and CD8^+^ T cells in the abscopal tumor were obtained after MWA treatment. **(G)** The percentages of CD4^+^ and CD8^+^ T cells in the axillary lymph node were also computed following MWA. **(H)** The percentage of CD11c^+^ DCs in the axillary lymph node from mice treated with MWA was determined. ns, p > 0.05; *p < 0.05; **p<0.01; ***p < 0.001.

To investigate the mechanism whereby MWA at 3 w–3 min activated the abscopal effect in CT26 tumor-bearing mice, flow cytometry was employed to analyze the immune microenvironment in the abscopal tumors, spleens, and axillary lymph nodes. Flow cytometric analysis on splenic CD4^+^ TNF-α^+^ or CD8 ^+^ TNF-α^+^ T cells has demonstrated that MWA treatment significantly elevated both populations (CD4^+^ TNF-α^+^ T cells, 1.407 ± 0.3415%) (CD8^+^ TNF-α^+^ T-cells, 1.057 ± 0.06658%) when compared with control (CD4^+^ TNF-α^+^ T cells, 0.6900 ± 0.0700%) (CD8^+^ TNF-α^+^ T cells, 0.3567 ± 0.1234%) (p < 0.05, **Figure 3A**) (p < 0.001, **Figure 3B**). The proportions of CD4^+^ (26.03 ± 3.014%) and CD8^+^ T cells (13.23 ± 4.561%) in the spleen was slightly increased following MWA treatments when compared with control (CD4^+^ T cells, 24.03 ± 3.980%) (CD8^+^ T cells, 10.17 ± 2.655%) (**Figure 3C**), although no statistical difference was observed. This implies that the immune response of the mice spleens triggered by MWA is relatively weak. To explore whether MWA primed T cells, flow cytometry was employed to examine the capacity of mouse T cells to secrete IFN-γ. Outcomes demonstrated that the MWA group activated anti-tumor immune response, and the proportion of intratumoral CD4^+^ IFN-γ^+^ T cells (1.640 ± 0.7686%) and CD8^+^ IFN-γ^+^ T cells (1.430 ± 0.1609%) in abscopal tumors from the MWA group were significantly increased when compared with the control group (CD4^+^ IFN-γ^+^ T cells 0.3533 ± 0.07506%) (CD8^+^ IFN-γ^+^ T cells 0.4867 ± 0.1172%) (p < 0.05, **Figure 3D**; p < 0.01, **Figure 3E**). Concerning the proportions of CD4^+^ and CD8^+^ T cells in the abscopal tumors, there is no significant difference between controls (CD4^+^ T cells, 4.540 ± 1.372%) (CD8^+^ T cells, 1.827 ± 0.8107%) and MWA treatments (CD4^+^ T cells, 5.870 ± 1.957%) (CD8^+^ T cells, 2.357 ± 0.7129%) (**Figure 3F**). Moreover, the immune microenvironment in the draining lymph nodes (axillary lymph nodes) was also identified, and it exhibits a decrease in CD4^+^ T cells but with an increase in CD8^+^ T cells following MWA treatments (p < 0.05, **Figure 3G**). CD11c^+^ DCs in the axillary lymph node from the MWA group showed a significant increase in CD86 expression (p < 0.01, **Figure 3H**), implying that CD11c^+^ DCs from mice in the MWA group had stronger antigen-presenting ability.

### 3.4 The mice presenting significant abscopal effect after MWA displayed stronger systemic immune activation

In order to explore the mechanism of the difference in induced abscopal effect in mice, the mouse models with primary and abscopal tumors were constructed again and randomly divided mice into the untreated group and the MWA group. We monitored the changes in the volume of abscopal tumor in mice and divided the MWA group into the more significant abscopal effect group and the abscopal effect group according to the slope of the growth curve.

**Figure 4:**
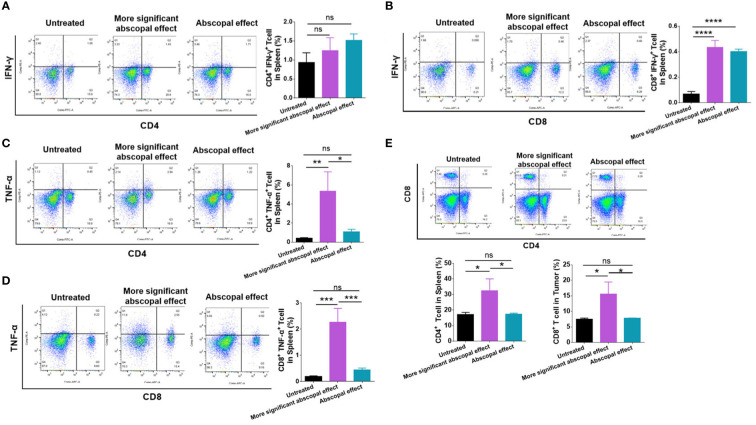
The mice presenting significant abscopal effect after MWA induced stronger systemic immune activation. The percentages of CD4^+^ IFN-γ^+^ T cells **(A)** and CD8^+^ IFN-γ^+^ T cells **(B)** in the spleen from mice presenting significant abscopal effect following MWA treatment were observed. The percentages of CD4^+^ TNF-α^+^ T-cells **(C)** and CD8^+^ TNF-α^+^ T-cells **(D)** in the spleen from mice presenting significant abscopal effect after MWA were 250 determined. **(E)** The percentages of CD4^+^ and CD8^+^ T cells in the spleen from mice presenting significant abscopal effect following MWA treatment were also identified. ns, p > 0.05; *p < 0.05; **p < 0.01. ***p < 0.001. ****p < 0.0001.

Immune cells in the spleen were examined by flow analysis to elucidate the difference in the abscopal effect in model mice. The proportion of CD4^+^ IFN-γ^+^ T cells (1.243% ± 0.3408%) showed a rising tendency after MWA compared with the control group (0.9333% ± 0.2558%) (**Figure 4A**), although no statistical difference was observed. The proportion of CD8^+^ IFN-γ^+^ T cells (0.4333 ± 0.05508%) was significantly higher in the more significant abscopal effect group than in the control group (0.06867% ± 0.01943%) (p < 0.0001, **Figure 4B**). The proportion of splenic CD4^+^ TNF-α^+^ T cells was higher in the more significant abscopal effect group (5.317 ± 2.060%) than that in the abscopal effect group (1.093 ± 0.2548%) (p < 0.05, **Figure 4C**); consistently, the proportion of splenic CD8^+^ TNF-α^+^ T cells was also elevated in more significant abscopal effect group (2.263 ± 0.5315%) than that in the abscopal effect group (0.4467 ± 0.06807%) (p < 0.001, **Figure 4D**). Furthermore, compared with the groups of control (CD4^+^ T cells, 17.20 ± 1.389%) (CD8^+^ T cells, 7.510 ± 0.3387%) or abscopal effect group (CD4^+^ T cells, 17.33 ± 0.7638%) (CD8^+^ T cells, 7.800 ± 0.07550%), mice in the group with more significant abscopal effect demonstrates higher T-cell proportion, with significant increases in both CD4^+^ (32.53 ± 7.564%) (p < 0.05, p < 0.05) and CD8^+^ T cells (15.53 ± 3.926%) (p < 0.05, p < 0.05) (**Figure 4E**), implying that MWA at 3 w–3 min stimulates systemic T-cell responses, specifically in the mice exhibiting significant abscopal effect.

### 3.5 The mice presenting significant abscopal effects after MWA had stronger intratumoral immune activation

**Figure 5:**
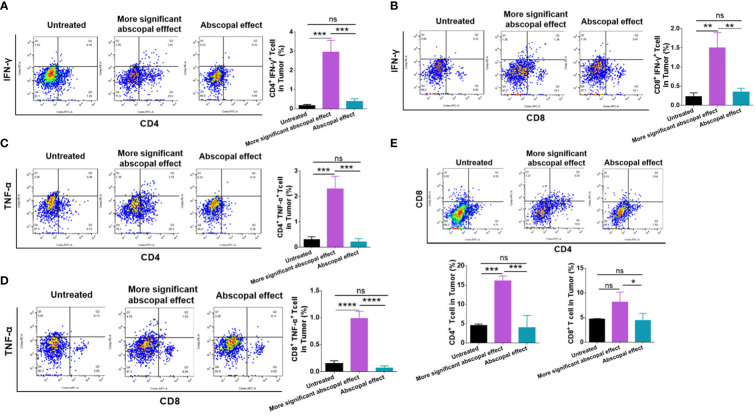
The mice presenting significant abscopal effects after MWA had stronger intratumoral immune activation. The percentages of CD4^+^ IFN-γ^+^ T cells **(A)** and CD8^+^ IFN-γ^+^ T cells **(B)** in the abscopal tumor were observed in mice presenting significant abscopal effect after MWA. The percentages of CD4^+^ TNF-α^+^ T cells **(C)** and CD8^+^ TNF-α^+^ T cells in the abscopal tumor from mice presenting significant abscopal effect after MWA were identified. **(E)** The percentages of CD4^+^ and CD8^+^ T cells in the abscopal tumor from mice presenting significant abscopal effect after MWA were both identified. ns, p > 0.05; *p < 0.05; **p < 0.01; ***p < 0.001; ****p < 0.0001.

Intratumoral immune cells were also examined by employing flow cytometry. The proportion of CD4^+^ IFN-γ^+^ T cells was significantly higher in the group exhibiting significant abscopal effect (2.950 ± 0.6219%) than that in the abscopal effect group (0.3900 ± 0.1300%) (p < 0.001, **Figure 5A**). Additionally, the proportion of CD8^+^ IFN-γ^+^ T cells in the more significant abscopal effect group (1.500 ± 0.3940%) was significantly higher than that in the abscopal effect group (0.3500 ± 0.09539%) (p < 0.01, **Figure 5B**). In comparison to the untreated or abscopal effect group, the mice presenting significant abscopal effect also elevated the proportion of CD4^+^ TNF-α^+^ T cells into the tumor (**Figure 5C**). A higher population of intratumoral CD4^+^ TNF-α^+^ T cells in the more significant abscopal effect group (2.303 ± 0.4899%) than in the abscopal effect group (0.2133 ± 0.1270%) was observed (p < 0.001, **Figure 5C**). In addition, the proportion of CD8^+^ TNF-α^+^ T cells demonstrated a similar tendency increasing in the mice exhibiting significant abscopal effect (0.9867 ± 0.1305%) compared with the abscopal effect group (0.07167 ± 0.03707%) (p < 0.0001, **Figure 5D**). The percentages of CD4^+^ and CD8^+^ T cells in the abscopal tumor were both increased in mice presenting significant abscopal effect following MWA compared with the abscopal effect group (**Figure 5E**), while only mice exhibiting significant abscopal effect showed higher percentages of intratumoral CD4^+^ T cells than the control group (p < 0.001, **Figure 5E**). These outcomes suggest that the mice presenting significant abscopal effect after MWA possessed stronger intratumoral immune activation.

## 4 Discussion

MWA can increase the local temperature of solid tumors and kill tumor cells (19). The procedure is less invasive, easy to operate, and can be applied repeatedly (20). Hence, it is frequently applied in patients who are intolerant to surgical excision and offers a means of treatment for patients with advanced tumors who have lost radical surgery (21). MWA has been extensively employed in the treatment of lung metastasis of colon cancer (22). The abscopal effect is considered to be the slowing or shrinking or disappearance of distant untreated lesions in tumor patients treated locally after the target lesion has subsided (23). We have discovered some cases of abscopal effect resulting from MWA in clinical works. Thus, for the first time, we evaluated whether MWA can trigger an abscopal effect in a CT26 mouse model of MSS colon cancer.

**Figure 6:**
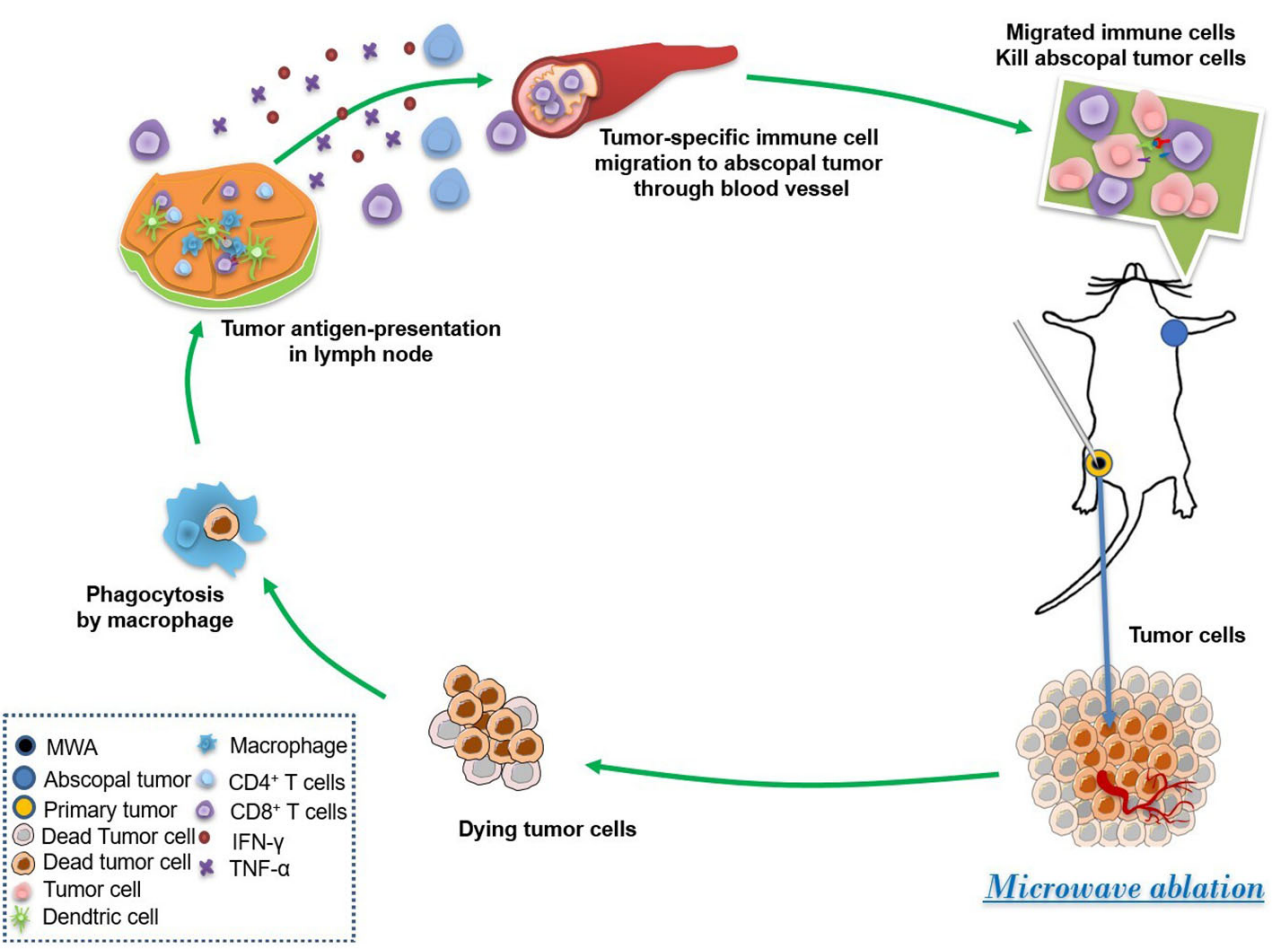
MWA induced the abscopal effect by the enhancement of systemic and local antitumor immunity. MWA begins the first step of the immune cycle by killing tumor cells and releasing tumor antigens. Then, dead tumor cells were phagocytosed by macrophages, and tumor antigen is shown by antigenic presenting cells such as DCs. Afterward, the tumor-specific immune cells are stimulated and migrated to the abscopal tumor through the blood vessel, finally suppressing the growth of the abscopal tumor.

MWA can inactivate tumors *in situ*, activate the immune system, and induce a specific immune response against tumors (24). Our research indicated that the MWA therapy reprogrammed the tumor microenvironment and suppressed the growth of abscopal tumors by the improvement of systemic antitumor immunity in CT26-bearing mice (**Figure 6**). MWA can make the local temperature of the tumor reach 60°C, which can cause protein degeneration and coagulation necrosis of tumor tissue, release a large amount of tumor-specific antigen, and initiate an adaptive immune response (25). The tumor cells that die after MWA are left *in situ* and can activate antigen-presenting cells (APC) *in vivo*. Activated cytotoxic T cells are key effector cells in the whole tumor immunity (25, 26).

Recent research has demonstrated that MWA alone activates local and systemic T-cell responses in hepatocellular carcinoma and osteosarcoma (27, 28). Dead tumor cells can be modified into *in situ* vaccines, which recruit APCs, namely, dendritic cells, and macrophages to enhance antigen processing and presentation. APCs induced CD8^+^ T cells to kill tumor cells, and more dead tumor cells further promote antitumor immunity as a loop, stimulating a strong and lasting specific killing effect of tumor cells (29, 30). The stimulated CD8^+^ T cells are the primary effector cells of tumor immunity (31). Our data depict that the mice after MWA increased the proportion of CD8^+^ T cells in the lymph node; the antigen-presenting capacity of DC cells also increased significantly, suggesting that MWA killed tumor cells to release tumor antigen and then promoted local and systemic antitumor immunity in CT26-bearing mice.

MWA stimulates the immediate necrosis of the tumor tissue, which can enhance the occurrence of the systemic inflammatory response (SIR) (32). Yu et al. discovered that CD8^+^ T cells could release IFN-γ and TNF-α after MWA (27), which was in line with our results. In our experiments, the high expression of inflammatory factor TNF-α has been observed in spleens and abscopal tumors of mice treated with MWA in CT26-bearing mice. This series of reaction processes may boost tumor cell necrosis, apoptosis, and immunogenic death and further stimulate innate and adaptive immune responses.

Th1 cells promote persistent tumor specific cytotoxic response and stimulate strong immune memory for tumor rechallenge (33). Th1 cells yield IL-2, IFN-γ, and TNF-α, which leads to cell-mediated immunity and phagocyte-dependent inflammation. IFN-γ secreted from CTLs is a cytokine that is essential for innate and adaptive immunity against cancer (34). The stimulation activated by MWA amplified the production of IFN-γ by Th1 cells in the abscopal tumors. These outcomes may show that MWA could initiate the Th1-type response and improve systemic tumor-specific immunity. Then, we discovered that MWA at 3 w–3 min differently induced abscopal effects in mice. Mice exhibiting significant abscopal effect following MWA significantly elevated the secretion of IFN-γ and TNF-α by T cells both in abscopal tumors and in spleens, implying that stronger systemic and intratumoral immunity was accomplished by MWA. Our experiments further validated that the stronger the immune activities by MWA, the more significant the abscopal effect induced in CT26-bearing mice.

When a probe is inserted into the tumor tissue for ablation, three distinct regions are formed based on the temperature: the center region undergoes coagulative necrosis at temperatures of 50°C or higher, the transition zone experiences sublethal heat-induced damage at temperatures between 41°C and 45°C, and the peripheral region receives an elevated supply of oxygen due to augmented blood flow, which may lead to the generation of reactive oxygen species and free radicals. When local tissues are damaged, hyaluronic acid and markers of endothelial damage become exposed. This stimulates the production of chemokines and vascular adhesion molecules, which in turn recruit immune cells to the affected site (35). In a clinical study on breast cancer patients, MWA was found to significantly induce increased levels of ICOS+ activated CD4^+^ T cells and serum IFN-γ, indicating a multifaceted impact on the local and systemic levels beyond coagulation necrosis and protein degeneration (36). However, the immune response induced by MWA is often weak and not sustainable due to the highly suppressive tumor microenvironment. TIGIT expression was found to be upregulated in various immune cells after MWA, and combining TIGIT blockade with MWA significantly enhanced the expansion and functionality of CD8^+^ tumor-infiltrating lymphocytes (TILs) and reshaped myeloid cells in the tumor microenvironment (37). Furthermore, blockade of PD-1/CTLA-4 or LAG3 enhanced the systemic antitumor immune response induced by MWA in preclinical mouse models (31, 38, 39). Thus, combining immune checkpoint inhibitors (ICIs) with MWA has emerged as a promising approach for achieving synergistic anti-tumor immunity.

The research has limitations, as it only used a murine CRC cell line and was not confirmed in other models. The metastasis model was triggered by subcutaneous inoculation instead of spontaneous metastasis in mice. The conclusion was only confirmed in the mouse model, which has limitations. Recently developed patient-derived organoids (PDOs) technology has been shown to replicate the tumor traits of patients and can help predict their response to treatment (40–42). Using patient-derived organoids (PDOs) and single-cell sequencing can help target specific populations and generalize immune profiles for determining the abscopal effect after MWA treatment.

## 5 Conclusions

Our research has screened combinations of ablation power and time for subcutaneous tumors in CT26-bearing mice and successfully shown that MWA at 3 w–3 min not only suppressed the growth of primary tumors but also triggered abscopal effects in mice with MSS colorectal cancer. Additional investigation of the mechanism has demonstrated that abscopal effects after MWA depend on the improvement of systemic and intratumoral antitumor immunity. Our research offers a theoretical basis for investigating the enhancement of the abscopal effect following MWA and for combinational treatments with MWA and immunotherapy.

## Data availability statement

The raw data supporting the conclusions of this article will be made available by the authors, without undue reservation.

## Ethics statement

The animal study was reviewed and approved by First Affiliated Hospital of Shandong First Medical University and Shandong Provincial Qianfoshan Hospital.

## Author contributions

LY performed the study and composed this manuscript, while HX and LW are responsible for drawing pictures. MC and JL were responsible for primary data generation and analysis. JX is involved in cell culture and animal rearing. XY and ZW are responsible for the revision of the paper. In addition, we have two corresponding authors in this manuscript. QX and JL contributed to the study design and manuscript revisions. All the authors contributed to the article and approved the submitted version.

## References

[1] Kurilova I Gonzalez-Aguirre A Beets-Tan RG Erinjeri J Petre EN Gonen M. Microwave ablation in the management of colorectal cancer pulmonary metastases. Cardiovasc Intervent Radiol (2018) 41(10):1530–44. doi: 10.1007/s00270-018-2000-6 PMC6944322 29845348

[2] Cheng G Shi L Qiang W Wu J Ji M Lu Q. The safety and efficacy of microwave ablation for the treatment of crc pulmonary metastases. Int J Hyperthermia (2018) 34(4):486–91. doi: 10.1080/02656736.2017.1366553 28847194

[3] Xu R Wang W Zhu B Lin X Ma D Zhu L. Disease characteristics and treatment patterns of Chinese patients with metastatic colorectal cancer: a retrospective study using medical records from China. BMC Cancer (2020) 20(1):131. doi: 10.1186/s12885-020-6557-5 32070312 PMC7029588

[4] Ferguson CD Luis CR Steinke K. Safety and efficacy of microwave ablation for medically inoperable colorectal pulmonary metastases: single-centre experience. J Med Imaging Radiat Oncol (2017) 61(2):243–9. doi: 10.1111/1754-9485.12600 28266145

[5] Kepp O Marabelle A Zitvogel L Kroemer G. Oncolysis without viruses - inducing systemic anticancer immune responses with local therapies. Nat Rev Clin Oncol (2020) 17(1):49–64. doi: 10.1038/s41571-019-0272-7 31595049

[6] Parmar A Chan KKW Ko YJ. Metastatic colorectal cancer: therapeutic options for treating refractory disease. Curr Oncol (2019) 26(Suppl 1):S24–32. doi: 10.3747/co.26.5575 PMC6878938 31819707

[7] Wright AS Lee FT Jr. Mahvi DM. Hepatic microwave ablation with multiple antennae results in synergistically larger zones of coagulation necrosis. Ann Surg Oncol (2003) 10(3):275–83. doi: 10.1245/aso.2003.03.045 12679313

[8] Macchi M Belfiore MP Floridi C Serra N Belfiore G Carmignani L. Radiofrequency versus microwave ablation for treatment of the lung tumours: lumira (Lung microwave radiofrequency) randomized trial. Med Oncol (2017) 34(5):96. doi: 10.1007/s12032-017-0946-x 28417355

[9] Belfiore G Ronza F Belfiore MP Serao N di Ronza G Grassi R. Patients' survival in lung malignancies treated by microwave ablation: our experience on 56 patients. Eur J Radiol (2013) 82(1):177–81. doi: 10.1016/j.ejrad.2012.08.024 23099201

[10] Mole RH. Whole body irradiation; radiobiology or medicine? Br J Radiol (1953) 26(305):234–41. doi: 10.1259/0007-1285-26-305-234 13042090

[11] Xing D Siva S Hanna GG. The abscopal effect of stereotactic radiotherapy and immunotherapy: fool's gold or El Dorado? Clin Oncol (Royal Coll Radiologists (Great Britain)) (2019) 31(7):432–43. doi: 10.1016/j.clon.2019.04.006 31005381

[12] Papaccio F Rosello S Huerta M Gambardella V Tarazona N Fleitas T. Neoadjuvant chemotherapy in locally advanced rectal cancer. Cancers (Basel) (2020) 12(12). doi: 10.3390/cancers12123611 PMC7761666 33287114

[13] Abdo J Cornell DL Mittal SK Agrawal DK. Immunotherapy plus cryotherapy: potential augmented abscopal effect for advanced cancers. Front Oncol (2018) 8:85. doi: 10.3389/fonc.2018.00085 29644213 PMC5882833

[14] Chen J Qian W Mu F Niu L Du D Xu K. The future of cryoablation: an abscopal effect. Cryobiology (2020) 97:1–4. doi: 10.1016/j.cryobiol.2020.02.010 32097610

[15] Xu H Sun W Kong Y Huang Y Wei Z Zhang L. Immune abscopal effect of microwave ablation for lung metastases of endometrial carcinoma. J Cancer Res Ther (2020) 16(7):1718–21. doi: 10.4103/jcrt.JCRT_1399_20 33565523

[16] Wei Z Yang X Ye X Huang G Li W Han X. Camrelizumab combined with microwave ablation improves the objective response rate in advanced non-small cell lung cancer. J Cancer Res Ther (2019) 15(7):1629–34. doi: 10.4103/jcrt.JCRT_990_19 31939448

[17] Zhou F Feng B Yu H Wang D Wang T Ma Y. Tumor microenvironment-activatable prodrug vesicles for nanoenabled cancer chemoimmunotherapy combining immunogenic cell death induction and Cd47 blockade. Adv Mater (2019) 31(14):e1805888. doi: 10.1002/adma.201805888 30762908

[18] Fu X Yang Y Xie J Pan X Yang X Du Z. Subcutaneous inoculation position affects the immune environment in Ct26 carcinomas. Biochem Biophys Res Commun (2019) 512(2):244–9. doi: 10.1016/j.bbrc.2019.03.042 30879760

[19] Vogl TJ Nour-Eldin NA Hammerstingl RM Panahi B Naguib NNN. Microwave ablation (Mwa): basics, technique and results in primary and metastatic liver neoplasms - review article. Rofo (2017) 189(11):1055–66. doi: 10.1055/s-0043-117410 28834968

[20] Izzo F Granata V Grassi R Fusco R Palaia R Delrio P. Radiofrequency ablation and microwave ablation in liver tumors: an update. Oncologist (2019) 24(10):e990–e1005. doi: 10.1634/theoncologist.2018-0337 31217342 PMC6795153

[21] Chan MV Huo YR Cao C Ridley L. Survival outcomes for surgical resection versus ct-guided percutaneous ablation for stage I non-small cell lung cancer (Nsclc): a systematic review and meta-analysis. Eur Radiol (2021) 31(7):5421–33. doi: 10.1007/s00330-020-07634-7 33449192

[22] Vogl TJ Nour-Eldin NA Albrecht MH Kaltenbach B Hohenforst-Schmidt W Lin H. Thermal ablation of lung tumors: focus on microwave ablation. Rofo (2017) 189(9):828–43. doi: 10.1055/s-0043-109010 28511267

[23] Ngwa W Irabor OC Schoenfeld JD Hesser J Demaria S Formenti SC. Using immunotherapy to boost the abscopal effect. Nat Rev Cancer (2018) 18(5):313–22. doi: 10.1038/nrc.2018.6 PMC5912991 29449659

[24] Dumolard L Ghelfi J Roth G Decaens T Macek Jilkova Z. Percutaneous ablation-induced immunomodulation in hepatocellular carcinoma. Int J Mol Sci (2020) 21(12). doi: 10.3390/ijms21124398 PMC7352237 32575734

[25] Chu KF Dupuy DE. Thermal ablation of tumours: biological mechanisms and advances in therapy. Nat Rev Cancer (2014) 14(3):199–208. doi: 10.1038/nrc3672 24561446

[26] Carrafiello G Lagana D Mangini M Fontana F Dionigi G Boni L. Microwave tumors ablation: principles, clinical applications and review of preliminary experiences. Int J Surg (2008) 6 Suppl 1:S65–9. doi: 10.1016/j.ijsu.2008.12.028 19186116

[27] Yu Z Geng J Zhang M Zhou Y Fan Q Chen J. Treatment of osteosarcoma with microwave thermal ablation to induce immunogenic cell death. Oncotarget (2014) 5(15):6526–39. doi: 10.18632/oncotarget.2310 PMC4171648 25153727

[28] Dong BW Zhang J Liang P Yu XL Su L Yu DJ. Sequential pathological and immunologic analysis of percutaneous microwave coagulation therapy of hepatocellular carcinoma. Int J Hyperthermia (2003) 19(2):119–33. doi: 10.1080/0265673021000017154 12623635

[29] Kroemer G Galluzzi L Kepp O Zitvogel L. Immunogenic cell death in cancer therapy. Annu Rev Immunol (2013) 31:51–72. doi: 10.1146/annurev-immunol-032712-100008 23157435

[30] Ahmed A Tait SWG. Targeting immunogenic cell death in cancer. Mol Oncol (2020) 14(12):2994–3006. doi: 10.1002/1878-0261.12851 33179413 PMC7718954

[31] Huang S Li T Chen Y Liu J Wang Y Yang C. Microwave ablation combined with anti-Pd-1 therapy enhances systemic antitumor immunity in a multitumor murine model of Hepa1-6. Int J Hyperthermia (2022) 39(1):278–86. doi: 10.1080/02656736.2022.2032406 35129044

[32] Erturk MS Cekic B Celik M. Microwave ablation of benign thyroid nodules: effects on systemic inflammatory response. J Coll Physicians Surg Pak (2020) 30(7):694–700. doi: 10.29271/jcpsp.2020.07.694 32811597

[33] Romagnani S. T-Cell subsets (Th1 versus Th2). Ann Allergy Asthma Immunol (2000) 85(1):9–21. doi: 10.1016/s1081-1206(10)62426-x 10923599

[34] Lin CF Lin CM Lee KY Wu SY Feng PH Chen KY. Escape from ifn-Gamma-Dependent immunosurveillance in tumorigenesis. J BioMed Sci (2017) 24(1):10. doi: 10.1186/s12929-017-0317-0 28143527 PMC5286687

[35] Yu L Cheng M Liu J Ye X Wei Z Xu J. Crosstalk between microwave ablation and ferroptosis: the next hot topic? Front Oncol (2023) 13:1099731. doi: 10.3389/fonc.2023.1099731 36712497 PMC9880492

[36] Zhou W Yu M Pan H Qiu W Wang H Qian M. Microwave ablation induces Th1-type immune response with activation of icos pathway in early-stage breast cancer. J Immunother Cancer (2021) 9(4). doi: 10.1136/jitc-2021-002343 PMC8021888 33795388

[37] Chen Y Huang H Li Y Xiao W Liu Y Chen R. Tigit blockade exerts synergistic effects on microwave ablation against cancer. Front Immunol (2022) 13:832230. doi: 10.3389/fimmu.2022.832230 35320940 PMC8935077

[38] Duan X Wang M Han X Ren J Huang G Ju S. Combined use of microwave ablation and cell immunotherapy induces nonspecific immunity of hepatocellular carcinoma model mice. Cell Cycle (2020) 19(24):3595–607. doi: 10.1080/15384101.2020.1853942 PMC7781627 33283623

[39] Shao D Chen Y Huang H Liu Y Chen J Zhu D. Lag3 blockade coordinates with microwave ablation to promote Cd8(+) T cell-mediated anti-tumor immunity. J Trans Med (2022) 20(1):433. doi: 10.1186/s12967-022-03646-7 PMC9524118 36180876

[40] Cabeza-Segura M Garcia-Mico B La Noce M Nicoletti GF Conti V Filippelli A. How organoids can improve personalized treatment in patients with gastro-esophageal tumors. Curr Opin Pharmacol (2023) 69:102348. doi: 10.1016/j.coph.2023.102348 36842387

[41] Papaccio F Garcia-Mico B Gimeno-Valiente F Cabeza-Segura M Gambardella V Gutierrez-Bravo MF. "Proteotranscriptomic analysis of advanced colorectal cancer patient derived organoids for drug sensitivity prediction". J Exp Clin Cancer Res (2023) 42(1):8. doi: 10.1186/s13046-022-02591-z 36604765 PMC9817273

[42] Papaccio F Cabeza-Segura M Garcia-Mico B Tarazona N Roda D Castillo J. Will organoids fill the gap towards functional precision medicine? J Pers Med (2022) 12(11). doi: 10.3390/jpm12111939 PMC9695811 36422115

