# Supplementary material for: Microwave ablation induces abscopal effect via enhanced systemic antitumor immunity in colorectal cancer
Source: Front Oncol. 2023 Apr 27;13:1174713. doi: 10.3389/fonc.2023.1174713 (PMC10174442; doi:10.3389/fonc.2023.1174713)
Supplement: Supplementary file 1 [file DataSheet_1.docx]

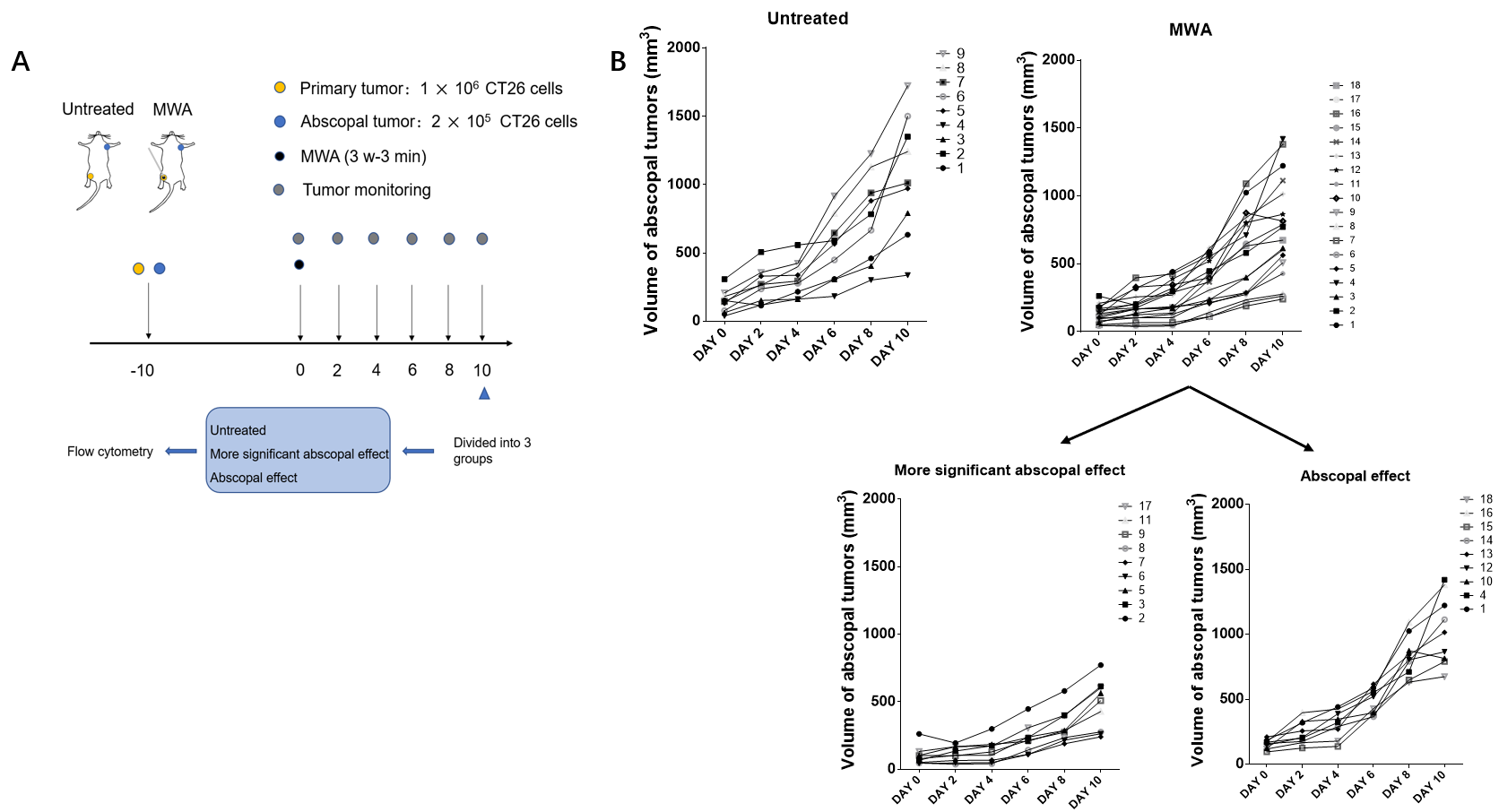


**Supplementary Fig. 1 (A)** Balb/c mice were inoculated with CT26 cells. When tumor size reached 250 mm^3^ in volume, CT26-bearing mice were treated with MWA (Untreated, n = 9; MWA, n = 18). **(B)** The growth of abscopal tumors in each mouse after MWA was shown during the observation period. Sort the slope of the abscopal tumor growth curve trendline for each mouse in the MWA group, and define the nine mice with smaller slopes as the more significant abscopal effect group (No. 2, 3, 5, 6, 7, 8, 9, 11, 17). Define the additional nine mice in the MWA group with larger slopes as the abscopal effect group (No. 1, 4, 10, 12, 13, 14, 15, 16,18).
